# Australian general practitioners’ perspectives on their role in well-child health care

**DOI:** 10.1186/1471-2296-14-2

**Published:** 2013-01-03

**Authors:** Adrian Jeyendra, Jeremy Rajadurai, Joanna Chanmugam, Alan Trieu, Suraj Nair, Radheshan Baskaran, Virginia Schmied

**Affiliations:** 1 Institutional Address: University of Western Sydney – School of Medicine, (Building 30), Goldsmsith Avenue, Campbelltown, NSW, 2560, Australia; 2 Institutional Address: University of Western Sydney – School of Nursing and Midwifery, Victoria Rd, Parramatta, NSW, 2150, Australia

**Keywords:** General practitioner, Well-child, Family, Role, Australian

## Abstract

**Background:**

In a General Practitioner (GP) setting, preventative medicine is reported as the predominant source of health care for the well-child. However, the role of the GP in well-child health care is not well understood in Australia. The aim of this study was to describe the role of the GP in providing services for well-children and families in Australia.

**Methods:**

This was a qualitative descriptive study. Face-to-face interviews were held with 23 GPs to identify their role in the provision of well-child health care. Participants worked in a variety of general practice settings and 21 of the 23 GPs worked in the Greater Western Sydney area.

**Results:**

Five main themes were identified in the analysis: ‘prevention is better than cure’, ‘health promotion: the key messages’, ‘working with families’, ‘working with other health professionals’, and ‘barriers to the delivery of well-child health services’.

**Conclusions:**

Participating GPs had a predominantly preventative focus, but in the main well-child care was opportunistic rather than proactive. The capacity to take a primary preventative approach to the health of children and families by GPs is limited by the increasing demands to manage chronic disease. Serious consideration should be given to developing collaborative models of care where GPs are joined up with services funded by State and Territory governments in Australia, such as the universal maternal child and family health nursing services that have well children and families as their prime focus.

## Background

The early years of a child’s life are crucial for optimising growth and development [1]. Despite the halving of child mortality rates in Australia over the past two decades [2], health outcomes for Australian children (particularly those from an Indigenous background) do not compare favourably internationally [3]. Australia has a well recognised system of universal child and family health services. Universal health services are those intended to be provided to all in the population because they are believed to confer benefits to children and families (e.g. antenatal care, child health services, school education). This contrasts with primary health care as the first point of contact in the health system where all those who need care can access it. Midwives provide care across pregnancy, birth and the postnatal period for up to six weeks after birth in some models. Child and family health nurses (also known as maternal and child health nurses) are registered nurses with specialist qualifications in child and family health who provide primary and secondary prevention services for families and children from birth to school entry [4]. General practitioners (GPs) also provide significant primary care services for children and families [5] and therefore they are in an ideal position to play a central role in the early detection of developmental and behavioural problems in children [6]. However, there is little published research on the role that GPs play in the provision of well child health services or how they collaborate with, complement or duplicate services and the educational preparation, competencies and skills required to deliver effective services.

The Royal Australian College of General Practitioners (RACGP) states that children should be screened opportunistically, to assess hearing, vision, language and social skills, as well as growth and development [7]. Further, the National Health and Medical Research Council (NHMRC) emphasise that GPs should work with parents, nurses and others in the community to maintain awareness of risk and protective factors that affect child and family wellbeing [8]. A significant development in Australia has been the introduction of the Healthy Kids Check, a checklist of assessments to gather health information, identify health problems and promote healthy lifestyles at four years of age [9]. The Medire item number (national health insurance) for this check, intended to identify children in need of additional services prior to school entry, does not appear to have been well utilised by GPs. Medicare data show that in the two years since its introduction in July 2008, only 81,463 Healthy Kids’ Checks have been done. That’s well below what was anticipated for a nation with some 260,000 four-year olds [10]. Most recently, the Australian federal government has announced a plan to use GP services to screen all three year old children for possible mental health problems. While supported by some early development specialists and mental health experts, this move has been widely criticised in Australia [11].

General practitioners therefore, have the potential to play a key role in preventative health care for children and families [12], yet very little is known about their role in promoting child health and development. The purpose of this paper is to describe the role of the GP in providing services for well-children and families, including if, and how, the GP works in collaboration with other professionals and the constraints encountered in meeting the needs of well-children and families.

For the purpose of this study, we have defined early childhood as the period between birth and eight years of age.

## Methods

### Study design

This was a qualitative descriptive study. Participating GPs were interviewed to explore their role in the provision of well-child care. Data were analysed using thematic analysis. Ethics approval for the study was obtained from the University of Western Sydney Human Research Ethics Committee. Each GP provided informed consent prior to the individual interviews.

### Setting and sample

The study was conducted in Sydney, NSW with 23 GPs who worked in a variety of general practice settings including solo practices and medical centres with up to 15 GPs. From the 25 GPs originally invited, two chose not to participate due to time constraints. Participants were approached based on the location of their practice, with many having an affiliation with University of Western Sydney. Twenty one participants worked in Sydney’s western suburbs. Equal numbers of male and female GPs participated (see Table 1). The majority of GPs interviewed were not Australian born, and spoke a language other than English. Most participants interviewed worked full-time. While 15 of the 23 GPs had more than 10 years of experience as a GP, only nine GPs had worked in their current practice for over 10 years. More than half the GPs had further education past the bachelor degree, with seven having training specific to child and family health (see Table 1).

**Table 1:** General practitioner demographics

**General background**	**n** (**%)**
**Sex**	
Male	11 (47.8)
Female	12 (52.2)
**Australian Born**	
Yes	1 (4.3)
No	22 (95.7)
**Speaks Language other than English**	
Yes	21 (91.3)
No	2 (8.7)
Education	
**Highest Education**	
Bachelor	8 (34.8)
Post Graduate Diploma	12 (52.2)
Masters	2 (8.7)
Doctorate	1 (4.3)
Specific Training in Child and Family Health*	
Yes	7 (30.4)
No	16 (69.6)

* Accepted diplomas in child health, family health or Obstetrics & Gynaecology.

### Data collection

Face-to-face interviews were conducted by authors 1 to 6 (fourth year medical students), with 23 GPs, either in the GPs’ practices or a location of their convenience. Key prompts were used to explore the role of the GP in child and family health (see Table 2). Training and support to conduct the interviews was provided by author 7. The interviews ranged from 15 to 25 minutes in duration, and were digitally recorded.

**Table 2:** Key prompts


1 Tell me about your role as a General Practitioner in working with well children and families.
2 What other services do you work with or collaborate with, in providing services/care for well-children and families?
3 Could you talk about the constraints in your practice for providing optimal care to children and families?
4 In the community where your practice is, what other services are available to enhance the health and wellbeing of children and families?
5 How do you see your role and the role of this general practice contributing to the universal health service system for child and family health?
6 There are a lot of changes happening with health reforms in Australia. From your perspective how can things be improved for children and families?

Key prompts used when interviewing GPs.

### Data analysis

Recorded interviews were transcribed verbatim. All team members read the transcripts and together undertook line by line coding of five transcripts to develop a set of preliminary themes. Team members then individually coded three to four of the remaining transcripts each. Themes were then refined by the group using an iterative process.

## Results

Five themes were identified in the data analysis, these were: ‘prevention is better than cure’, ‘health promotion: the key messages’, ‘working with families’, ‘working with other health professionals’, and ‘barriers to the delivery of well-child health service’.

### Prevention is better than cure

The majority of GPs highlighted the importance of prevention; one stated “*prevention is better than cure*” (GP18), another prioritised preventative health stating “*first of all, we provide preventative medicine for the children…*” (GP5) and a third participant described that GPs “…*deal with normal GP things like immunisation, preventative medicine, growing and all the usual paediatric things*” (GP10). Another participant emphasised that a preventative approach *“…also covers not just the child, but the parents”* (GP9).

Some reported that they take a proactive approach to health promotion, wanting to “*catch things early*” (GP6). They achieved this through organising routine health checks to identify problems with development, as well as applying recall systems for immunisation to *“…make sure you get them back if they don’t come in time*” (GP7).

The majority however, described their role in child health as ‘opportunistic’, with consultations seen as an opportunity to conduct routine health checks and ensure normal development,

"…so I don’t just give them a needle, I also talk about development and what to expect as well (GP1),"

"We see them at four years old for their vaccinations, and then we do the health check then as well (GP20)."

A small number of participants were more focussed on children with an acute illness. When asked about their role in health care for children, they immediately reflected on the sick child rather than the well-child, some responding with, *“We normally see unwell children”* (GP6) and *“With well-children, they hardly come to the surgery. They normally come with a problem”* (GP11)*.*

### Health promotion: the key messages

While the participants spoke broadly about their role in health promotion, it appeared that this was focused on three areas, namely immunisation, breastfeeding and parent-infant relationships,

"Well, in the well-child, it would basically be, number one to keep them well – that is prevention, meaning immunisation, and preferably full immunisation (GP21)."

"You counsel the mother to see if they are breastfeeding or bottle feeding and how they are going with it, and if they are developmentally consistent (GP2)."

The participants demonstrated some familiarity with anticipatory guidance but provided limited illustrations of putting this into practice other than relating it to the use of the ‘blue book’ (personal health record) in easing parental concerns about their child’s development,

"The parents usually read it and before they reach the age, the parents should know at what age the child should be sitting up and what age they should be talking and how many words they should be talking sentences (GP2)."

Overall the approach to health promotion tended to be reactive, as the GP “…*would only see the child opportunistically, when the parent brings the child in…for immunisation*” (GP21). In response to conducting routine health checks, one GP answered, “*Normally when we do it, a motivated mother asks me, rather than doing it routinely*” (GP10). Some GPs agreed that it is “*Usually the parents* [who] *promote the issues to be checked with the children”* (GP12) rather than the GPs themselves.

### Working with families

Participants were also asked to describe how they worked with families. GPs identified their role as being educators and providers of support, *“I think in the early years of parenting it is more important to give them support”* (GP2). Providing information in a reassuring way was necessary to ensure that families were not distressed,

"We need to educate, we need to enhance, we need to give more information in a compassionate, empathetic way, but not to scare them (GP5)."

The importance of developing a relationship with parents was also emphasised, with one GP commenting that “…*developing a strong rapport and relationship with the family is the most important thing”* (GP14). Difficulty in maintaining continuity of care was considered a barrier for working effectively with families. Participants believed that lack of continuity occurred due to both GP and family factors,

"I work 3 days a week in my practice so sometimes I’d like to follow up a child, but it’ll be a day that I’m not there and they end up seeing a different GP so sometimes you tend to lose that follow up (GP3)."

This fragmented care was thought to result from switching doctors frequently,

"Here the family chooses the GP whenever they want, whoever they want. So they can move from GP to GP. So there can be a problem in the continuity of care (GP7)."

Those who were involved in shared antenatal care believed that they had the best opportunity for forming relationships with families from the outset. According to one GP, *“It’s easy with shared care, because most of them like it and they are more comfortable with us, because they have known us for a long time*” (GP13) and *“…the role of this for a GP is absolutely vital because it is the interface between the hospital and primary care”* (GP14).

Participants described the educational role of GPs including assisting families to interpret health information, as *“providing medical information in lay person terms is of paramount importance”* (GP5). They also reported that GPs need to help interpret information available from other sources, *“…a lot of the time, parents do their own research and ask us about that service”* (GP1). At times, however, this proved to be a challenge,

"The revolution of knowledge through the internet in one way its good but in another way it puts a lot of strain on us, because whenever we make a recommendation, they just Google it or research it and come back with a lot of queries or questions (GP10)."

### Working with other health professionals

All participants had some understanding of the services available for the well-child and families. However, only 15 out of the 23 GPs described some form of interaction with other services, which usually only amounted to referrals, *“If there is a problem then we’ll refer them to the appropriate services”* (GP11). These services included paediatricians, child psychologists and allied health services such as speech therapy, occupational therapy, social work, and audiologists. Some GPs also liaised with non-referral based local government services such as community and childcare groups to support children and families. In the main, GPs would work in collaboration with either a practice nurse or a community-based child and family health nurse in providing care for the well-child, through anticipatory guidance and support as well as health promotion services such as immunisation and information on feeding issues,

"A practice nurse is actually a big help…[they] give vaccinations, weigh the child and take measurements…as well as provide advice to parents (GP20)"

"In our practice, the nurses do the routine health checks. (GP4)"

Of the eight GPs who did not have any involvement with other services, the majority cited the redundancy of these services in well-child care, “*If both the child and family are well there isn’t much need to work with other services”* (GP14) and commented on logistical issues such as waiting times and poor communication, *“Really the problem is the lack of communication between the* [specialist] *services and the GPs*” (GP17).

### Barriers to the provision child and family health

The major limitation that GPs identified was finding the time to spend in consultations to provide optimal well-child care. Despite this, one participant added, *“In my practice I don’t think time can be an excuse. You can always find the time”* (GP11).

Another issue faced by GPs in providing optimal well-child care was the financial status of families, especially in practices that did not bulk bill, *“…some people can’t afford to even cover that [Medicare] gap, in the lower socioeconomic groups”* (GP8). Furthermore, some GPs identified the lack of knowledge of, and access to, services available for children under their care, *“Part of the problem is actually knowing what’s around, you can’t access them”* (GP9).

Nonetheless, the GPs recommended possible improvements to facilitate optimal well-child management. Improvements in communication between GPs and other health services were identified as important, both to increase quality of care as well as efficiency, *“Better information interflow between hospital and GPs is necessary…awareness of what services are available rather than duplicating some and lacking others”* (GP16). Participants also suggested increasing the number of health services in the community, *“…more community based services, especially community based nursing. Also easier access to allied health services, like speech pathologists”* (GP1).

## Discussion

The purpose of this study was to describe GPs’ perceptions of their role in the provision of well-child health care. As reported in the literature [6], most participants believed they had an important role in delivering preventative services to well-children and their families, including immunisations, assessing growth and development, discussing behavioural issues and providing information to parents regarding their concerns.

The majority of participants prioritised prevention however their approach to this varied. GPs who wanted to *“catch things early”* demonstrated a proactive approach to well-child health care. As reported in the literature [13], having a recall system in place to identify children ‘at risk’, for example those overdue for immunisation, is a strategy which has been shown to improve overall immunisation rates.

Most participants took an opportunistic approach to preventative health care, providing child health surveillance focused on growth, hearing, vision and speech when a child was brought in for a scheduled immunisation visit, an episodic illness or when brought in by a *“motivated mother”.* Arguably this is the most common approach in general practice [14] and is supported by practice guidelines [7].

The introduction of the Medicare item number for the four year old ‘Healthy Kids Check’ has provided another opportunity for GPs to undertake developmental assessment of children. It was introduced by the Australian government in July 2008 to “promote early detection of lifestyle risk factors, delayed development and illness, and introduce guidance for healthy lifestyles and early intervention strategies” [15].

Despite the weak evidence on the Healthy Kids Check, refinements could be made to better address current shortfalls in well-child healthcare. Therefore, more emphasis should be placed on screening for issues with a strong evidence base, including oral health and fluoride exposure, as opposed to issues not supported by evidence such as questioning toilet habits [16]. Furthermore, it appears that there has not been a good uptake of the four year check by GPs or by parents, with only 16% of four year olds receiving it in the first year of the scheme [17].

As reported by others, [18,19], while the GPs in this study believed they had an important role in disseminating key health promotion messages, in the main this was limited to immunisation, promoting breastfeeding and less often, addressing issues related to the parent–child relationship. The role of Australian GPs in promoting breastfeeding is not well understood. However, international studies demonstrate that routine preventative postnatal visits with primary care physicians can significantly improve breastfeeding duration [20]. The participating GPs did not demonstrate a clear understanding of anticipatory guidance. Research indicates that guidance about parent-infant interaction, sleep patterns, injury prevention, and reading at home during early childhood is associated with improved child and family functioning [21]. However, research also suggests that there are numerous missed opportunities for anticipatory guidance during well-child care and evidence that parents would like to be provided with more information [22,23].

While the participants also reported having an educative role, this was often described as ‘teaching’ and interpreting information for parents. The preventative care guidelines for general practice in Australia advocate the provision of parent education, including accident and injury prevention, nutrition advice and child health surveillance [7]. However, if this is done in a didactic way where the GP is ‘telling’ the family what to do, it is not likely to be successful [24]. This change from ‘passive recipient to active consumer’ can also result in the GP feeling that their role as a primary health care and information provider has diminished [25].

Several GPs identified developing a rapport or building a relationship as essential in providing continuity of care to well-children and their families. This is considered to be the core of general practice and is well supported in the literature [26]. However, they also reported that maintaining this relationship is limited and potentially threatened by parents frequently changing doctors, resulting in fragmented care.

Collaboration is important to achieve the goal of effective health care for children and families and requires health professionals from different disciplines and sectors to provide services in new ways. Collaboration exists on a continuum; with no communication between professionals at one end, moving to referrals with limited communication and ultimately to service integration at the other [27,28]. Evidence from the literature emphasises a need for health professionals to understand and respect each others’ skills and opinions in order to promote professional engagement and collaboration [29]. Therefore, interdisciplinary care becomes key to improving health outcomes, possibly highlighting the need for families with young children to have more contact with an appropriate health professional, such as Child and Family Health Nurses and not necessarily the GP. There are calls for collaboration and service integration to support children and families in the most useful way pedicle [4]. However, partnerships and service integration require time and funding to become embedded in clinical practice, and for many GPs, this goes no further than rudimentary referral based communication with little to no correspondence back [30].

The participating GPs indicated that they would prefer more feedback from services they referred their patients to. A proposed initiative by Nicolson et al. in resolving this barrier to well-child care is to implement a liaison position to be accountable for the communication outcomes [31]. Case conferencing and team care arrangements are further potential solutions to this constraint as it offers increased opportunities for GPs and other service professionals to work collectively in developing a consensus on how to best manage patients.

Furthermore, Gardner argued that while effective communication is pertinent in delivering services, it is equally important to have access to and awareness of a variety of available services [32].

The key limitation in GP provision of care for the well-child is lack of time [33]. GPs find it difficult to allocate adequate time to properly assess the well-child, as well as promote key health messages to parents. These time constraints are further aggravated by the “*crowding out*” of non-acute and preventive care visits by the primary care needs of Australia’s ageing population [34]. This may be alleviated by GPs forming stronger relationships with other health professionals, for example, Child and Family Health Nurses, who can provide comprehensive primary and secondary preventative services which include developmental assessment, health information and health promotion for such issues as breastfeeding, nutrition, dental care and immunisations as well as support for the emotional and social needs of families [4,35,36]

Finally, it is evident that may GPs defer the healthy kids check and other child health activities to practice nurses [37,38]. Practice Nursing is currently the fastest growing area in the health arena, with a 15% increase in the speciality between 2007 and 2009 and approximately 60% of all general practices including a practice nurse in the team [39]. Both Walsh and Barnes [38] and Denney-Wilson and colleagues [37] have recently surveyed practice nurses about their experience of conducting the Healthy Kids Check and working with children. Walsh and Barnes [38] found that practice nurses acknowledge that they lack skills in well child health care and are requesting increased education to be able to meet the demands placed on them. Nurses responding the survey by Denney-Wilson also reported that many parents are suspicious that the check implies a criticism of their parenting and so they are reluctant to attend. Other parents find it difficult to fit in between work and caring for the rest of the family. Further research on the role of practice nurses in providing well child health care is required.

### Limitations

This is a small qualitative study conducted in a specific metropolitan location and the findings cannot be generalised. In addition, interviews were limited by availability of GPs due to time constraints in busy practices.

Further research is needed with a larger sample of GPs to understand the role of GPs and how these services interact with the role of other health services, including Non Government Organisations, across universal and targeted services. Looking at parents’ perspectives in delivery of health services may also prove useful in identifying shortfalls and areas for future improvement.

## Conclusion

This study contributes to our understanding of GP perspective of their role in well-child care and is timely given the current health reforms in Australia such as Medicare Locals. Participating GPs had a predominantly preventative focus, but in the main well-child care was opportunistic rather than proactive. The findings suggest that GPs may require some professional development to support their role in working effectively with families and children to address missed opportunities in preventative care. Looking at parents’ perspectives in the delivery of health services would be useful in identifying shortfalls and areas for future improvement.

## Competing interest

None identified. We confirm that the content of this paper is currently not submitted or published in full or in part elsewhere.

## Authors’ contributions

AJ, JR, JC, AT, RB and SN carried out interviews with General Practitioners, thematically analysed the data and collaboratively wrote the manuscript. AJ and JR edited the manuscript for submission. VS conceived the study, and supervised its design and data analysis. All authors read and approved the final manuscript.

## Pre-publication history

The pre-publication history for this paper can be accessed here:

http://www.biomedcentral.com/1471-2296/14/2/prepub
